# Consistent inter‐individual differences in common marmosets (*Callithrix jacchus*) in Boldness‐Shyness, Stress‐Activity, and Exploration‐Avoidance

**DOI:** 10.1002/ajp.22566

**Published:** 2016-06-10

**Authors:** Vedrana Šlipogor, Tina Gunhold‐de Oliveira, Zoran Tadić, Jorg J.M. Massen, Thomas Bugnyar

**Affiliations:** ^1^ Department of Cognitive BiologyUniversity of ViennaViennaAustria; ^2^ Division of BiologyUniversity of ZagrebZagrebCroatia

**Keywords:** animal personality, consistent inter‐individual differences, non‐human primates, common marmosets, solitary setting, group personality

## Abstract

The study of animal personality, defined as consistent inter‐individual differences in correlated behavioral traits stable throughout time and/or contexts, has recently become one of the fastest growing areas in animal biology, with study species ranging from insects to non‐human primates. The latter have, however, only occasionally been tested with standardized experiments. Instead their personality has usually been assessed using questionnaires. Therefore, this study aimed to test 21 common marmosets (*Callithrix jacchus*) living in three family groups, in five different experiments, and their corresponding controls. We found that behavioral differences between our animals were not only consistent over time, but also across different contexts. Moreover, the consistent behaviors formed a construct of four major non‐social personality components: Boldness‐Shyness in Foraging, Boldness‐Shyness in Predation, Stress‐Activity, and Exploration‐Avoidance. We found no sex or age differences in these components, but our results did reveal differences in Exploration‐Avoidance between the three family groups. As social environment can have a large influence on behavior of individuals, our results may suggest group‐level similarity in personality (i.e., “group personality”) in common marmosets, a species living in highly cohesive social groups. Am. J. Primatol. 78:961–973, 2016. © 2016 The Authors. *American Journal of Primatology* published by Wiley Periodicals, Inc.

## INTRODUCTION

The psychology of personality in humans has been well established for more than a century [Galton, 1883], but surprisingly, consistent inter‐individual differences in animals were treated as noise and were, with some exceptions [Hebb, 1946], largely neglected up until 3 decades ago [e.g., Huntingford, 1976; for reviews please see Carere & Maestripieri, 2013; Koolhaas et al., 2010; Nettle & Penke, 2010; Réale et al., 2007]. Since then researchers have realized that personality variation is an important component of biological diversity [Smith & Blumstein, 2013] and highly relevant to evolution [Wolf et al., 2007]. Researchers from various fields in biology and psychology (behavioral ecology, comparative psychology, genetics, neuroendocrinology, development, evolution) studying species ranging from invertebrates such as octopuses to non‐human primates such as chimpanzees shifted their attention to personality [for reviews see Bell et al., 2009; Bergmüller & Taborsky, 2010; Bouchard & Loehlin, 2001; Dall & Griffith, 2014; Gosling, 2001; Koski, 2014; Réale et al., 2010; Stamps & Groothuis, 2010]. By definition, animal personalities are consistent ways in which animals of the same species differ in their behavior, over time and/or across different situations and/or contexts [Gosling, 2001; Réale et al., 2007, 2010; Sih et al., 2004]. For non‐human animals, five major axes of continuous personality traits have been suggested, the first three being non‐social ones (as they do not necessarily include the presence of a conspecific) and the last two being social ones (as they are connected to the presence or absence of conspecifics): Boldness‐Shyness (reaction to any risky situation, e.g., predators in a non‐novel situation), Exploration‐Avoidance (reaction to a new situation, e.g., environment, food, or object), Activity (the level of activity in a non‐novel environment), Aggressiveness (aggressive reaction to a conspecific) and Sociability (reaction of an animal to the presence or absence of a conspecific) [Réale et al., 2007]. These traits are sometimes investigated in a social setting, namely with other conspecifics (in dyads, subgroups, or whole groups) [e.g., Fairbanks, 2001; Koski & Burkart, 2015; Massen et al., 2013], and sometimes in a solitary setting (individually, e.g., Dammhahn, 2012; Dingemanse et al., 2002; Koski & Burkart, 2015; this study). Although these five traits are usually considered as standard personality traits, using a more bottom‐up approach includes the possibility that additional behavioral axes can also be part of personality [Koski, 2014].

Most primates live in highly complex social systems consisting of short‐ and long‐term social bonds and networks of interactions (affiliative or agonistic relationships, kinship, dominance hierarchies, alliances, etc.), and have a very rich behavioral repertoire [Chapais, 2001; Massen et al., 2010; Seyfarth & Cheney, 2012; Silk, 2007]. In such animals, personality could influence many aspects of daily life, for example group composition, group stability, social networks, individual behavior, dispersal, fitness, and so on, as has been shown in many taxa [Coleman, 2012; Croft et al., 2004; Krause et al., 2010; Massen & Koski, 2014; Seyfarth et al., 2014; Smith & Blumstein, 2008]. Although there have been some terminological and methodological discrepancies in measuring personality traits across different taxonomic levels [Carter et al., 2013], researchers of non‐human primates have assessed personality with one of the three following methods so far: personality ratings of individuals via questionnaires, behavioral measurements/ratings in the animals’ home environment or behavioral measurements in a series of standardized personality tests [Freeman et al., 2011; Stevenson‐Hinde et al., 1980b; Uher & Asendorpf, 2008; Weinstein et al., 2008].

In the first method, researchers take two substantially different approaches. In one approach, personality is assessed using a so‐called “five‐factor model” (FFM) [Digman, 1990] accompanied by questionnaires adapted from the human personality psychology [King & Figueredo, 1997; Weiss et al., 2006]. Here, human observers (i.e., animal caretakers or researchers) fill out species‐specific versions of questionnaires that typically contain a series of descriptive adjectives and their explanations. Each animal is rated on a five‐ or seven‐point (Likert) scale based on how well the adjective reflects its personal characteristics and personality scores are calculated from these values [Gosling, 2001]. These scores are then clustered in the five personality traits that follow from the human personality literature (aka “The Big Five”: Costa & McCrae [1992]), i.e., Agreeableness (A), Conscientiousness (C), Extraversion (E), Neuroticism (N), and Openness to Experience (O) [Digman, 1990]. As this approach uses different pre‐defined axes, comparative research that aims at understanding the evolution of personality traits in different animal lineages is limited. In the other approach, researchers use a more bottom‐up procedure to determine how adjectives from the questionnaires are grouped together into factors for the species of interest [Uher, 2008], which allows a better understanding of personality across different animal taxa [e.g., Capitanio, 1999, 2004; Capitanio & Widaman, 2005; McGuire et al., 1994; Stevenson‐Hinde & Zunz, 1978; Stevenson‐Hinde et al., 1980a,1980b; Uher, 2011a,2011b; Uher et al., 2013b]. Surprisingly, across research groups and model species, a degree of consistency in major dimensions of personality has been found, including, but not limited to, Confidence/Aggressiveness, Sociability, Excitability/Reactivity, and Equability [cf. Capitanio & Widaman 2005; Capitanio, 2004; Gosling, 2001].

The second method used by non‐human primate researchers relies on more traditional ethological methods and assesses personality through recordings of different behaviors that animals exhibit in daily (social) life, either in the wild or in their home enclosures in captivity. This method focuses on those behaviors that are commonly found in a species’ behavioral repertoire [Capitanio & Widaman, 2005; Koski, 2011; Rouff et al., 2005; Seyfarth et al., 2012; Sussman et al., 2013; Uher et al., 2013b] and can be regularly collected via focal protocols. Using this method, researchers have recently found that, similar to most other animals, primates show consistent inter‐individual differences [Koski, 2014] regarding Boldness (i.e., Boldness‐Shyness) [Rouff et al., 2005], Activity [Koski, 2011], and Anxiety (stress‐related behavior) [Koski, 2011]. Additionally, these studies found consistent inter‐individual differences in social personality traits, that is, in Sociability [Koski, 2011; Rouff et al., 2005] and Aggressiveness [Rouff et al., 2005], but also in some previously unreported social traits, for example Grooming‐Equitability and Positive Affect [chimpanzees, *Pan troglodytes*: Koski, 2011]. One drawback of this method, however, is that it focuses on common behaviors and might overlook animals’ reactions to rare occurrences that might also reflect personality, for example reactions to predators or novel objects/environments (i.e., Boldness‐Shyness & Exploration‐Avoidance). Also, this method is to some extent limited by the fact that individuals are usually tested in a group setting, which might be a confounding factor in achieving individual scores that are not influenced by group dynamics [but see Koski, 2011].

To overcome this problem the third method aims at gathering personality information that is rarely observed in daily life, using sets of standardized tests. Typically, these tests contain either a degree of novelty, for example a novel object/food, a frightening stimulus such as a predator, or an altered social environment, for instance a solitary or a group condition (i.e., different social setting). All behaviors (latencies, frequencies, and durations) are recorded during a fixed time period on two or more occasions. Afterwards, consistency across time, contexts and situations can be quantitatively measured, which makes this method reliable and reasonably objective, and also allows cross‐species comparisons. To date, several non‐human primate studies have used this approach to assess personality [Capitanio, 1999; Capitanio et al., 2012; Carter et al., 2012; Dammhahn, 2012; Fairbanks, 2001; Hebb, 1946; Koski & Burkart, 2015; Massen et al., 2013; Schneider et al., 1991; Stevenson‐Hinde et al., 1980b; Uher et al., 2008, 2013a]. For instance, Massen and colleagues [2013] tested 29 adult chimpanzees in a group setting in a battery of ten experiments. They found two different personality axes, namely Exploration‐Persistence and Boldness. Similar results emerged from a study that tested 117 gray mouse lemurs in two tests (novel object and open field) over a 3‐year period [Dammhahn, 2012]. Lemurs exhibited consistent inter‐individual variation and intra‐individual consistency in Boldness, Exploration, and Activity. Another study by Uher and colleagues [2008] tested four great ape species in a number of experimental tasks and found high temporal consistency in behaviors and low cross‐situational consistency in responses (before feeding and afternoon conditions).

Callitrichids represent the smallest primates, which makes them vulnerable to predation from raptor birds and snakes [Grzimek, 2003], careful with novel objects and spaces [Fragaszy & Visalberghi, 2004], and thus, a particularly interesting species for studying the non‐social personality axes. Previous studies noted that individual common marmosets (*Callithrix jacchus*) differ in their reactions to various stimuli, and that this is consistent within an individual, over time [Gunhold et al., 2015]. Indeed, Koski & Burkart [2015] have recently found experimental evidence for personality in this species. The animals were tested for Boldness, Exploration, and Persistence in a social setting in a battery of eight tests. Two experiments from this test battery were conducted again a year after the initial testing, but in a solitary setting and only once per individual and test. The consistent behaviors that emerged from this study formed two independent constructs: Boldness and Exploration. The authors found that both constructs were influenced by other group members in a social condition, resulting in a long‐term effect of group‐level similarity in personality. Additionally, whereas Boldness scores showed high consistency across solitary and social conditions, there were inconsistencies in Exploration scores between these two conditions, suggesting that these marmosets showed short‐term plasticity based on social influences in Exploration.

Note that, unlike in the social setting, Koski & Burkart's study [2015] does not provide experimental evidence for personality in the solitary setting, as the solitary condition was only conducted once per individual. Thus, the monkeys were not re‐tested to account for the repeatability of behavioral measurements. Although testing gregarious animals in a social setting is sensible because a social environment depicts normal behavior well [Koski, 2011], ecologically relevant arguments can be made why they should be also tested individually. On one hand, animals do not always encounter several possible daily life challenges like predation events or novel food as a group; on the other hand, repeated social interactions often modify (i.e., hinder through conformity or accentuate through facilitation) the expression of individual behavioral traits as found in dominance hierarchies and mating opportunities [Crockford et al., 2007; Webster & Ward, 2011]. Hence, it is very likely that the picture obtained by testing animals solely in a social setting is not complete [see also Koski & Burkart, 2015]. Furthermore, as most studies on non‐primates were conducted in a solitary setting, comparative research with studies in a social setting remains difficult.

In this study, we aimed to assess inter‐individual differences of common marmosets (*Callithrix jacchus*) in standardized repeated individual tests that, to our knowledge, have not been applied to marmosets yet. Specifically, we confronted captive marmosets twice (to test for repeatability) and in a solitary setting with five different experiments: (i) General Activity; (ii) Novel Food; (iii) Novel Object; (iv) Predator; and (v) Foraging Under Risk. Additionally, we designed all experiments with corresponding controls, as previous studies have raised the issue of the importance of controls in animal personality research [Carter, 2013] (see Methods and Results sections of this article and SEM for further details on controls). As our subjects were tested in experiments designed to capture non‐social personality traits, we hypothesized that these behaviors may form clusters of non‐social personality traits, namely Boldness‐Shyness, Exploration‐Avoidance, and Activity.

## METHODS

### Subjects

We tested 21 common marmosets (*Callithrix jacchus*) (12 males, 9 females) born in captivity and housed in three different family groups at the Department of Cognitive Biology, University of Vienna, Austria. Each group lived in an indoor cage (250 × 250 × 250 cm) of wire mesh connected to an outdoor cage (250 × 250 × 250 cm), and an experimental cage (146 × 36 × 110 cm) via a passageway system of tunnels with moveable doors. Each home enclosure contained wood shavings as floor bedding material and had plenty of enrichment objects (branches, ropes, platforms, blankets, sleeping boxes, tunnels). Visual contact between the family groups was prevented by an opaque plastic barrier between the adjacent cages, while acoustic and olfactory contact was possible. Temperature was maintained at 24–26°C at all times, and humidity was kept at 40–60%. Daylight was the main source of lighting, but additional lamps were placed above the enclosures to provide additional light to the animals in winter, and consequently they were maintained on a stable 12:12 hr light:dark cycle. Heating lamps were always available at certain places on top of each enclosure. The animals were fed daily at noon with a selection of different fruits, vegetables, grains, milk products, pellets, marmoset jelly, protein and vitamin supplements, and insects. Water was provided ad libitum. The housing conditions were in accordance with Austrian legislation and the European Association of Zoos and Aquaria (EAZA) husbandry guidelines for Callitrichidae. The research complied with protocols approved by the institutional board for animal experimentation (license number 2014‐016) and adhered to the legal requirements of Austria. The study also adhered to the American Society of Primatologists’ principles for the ethical treatment of primates.

### Experimental Design

**Figure 1:**
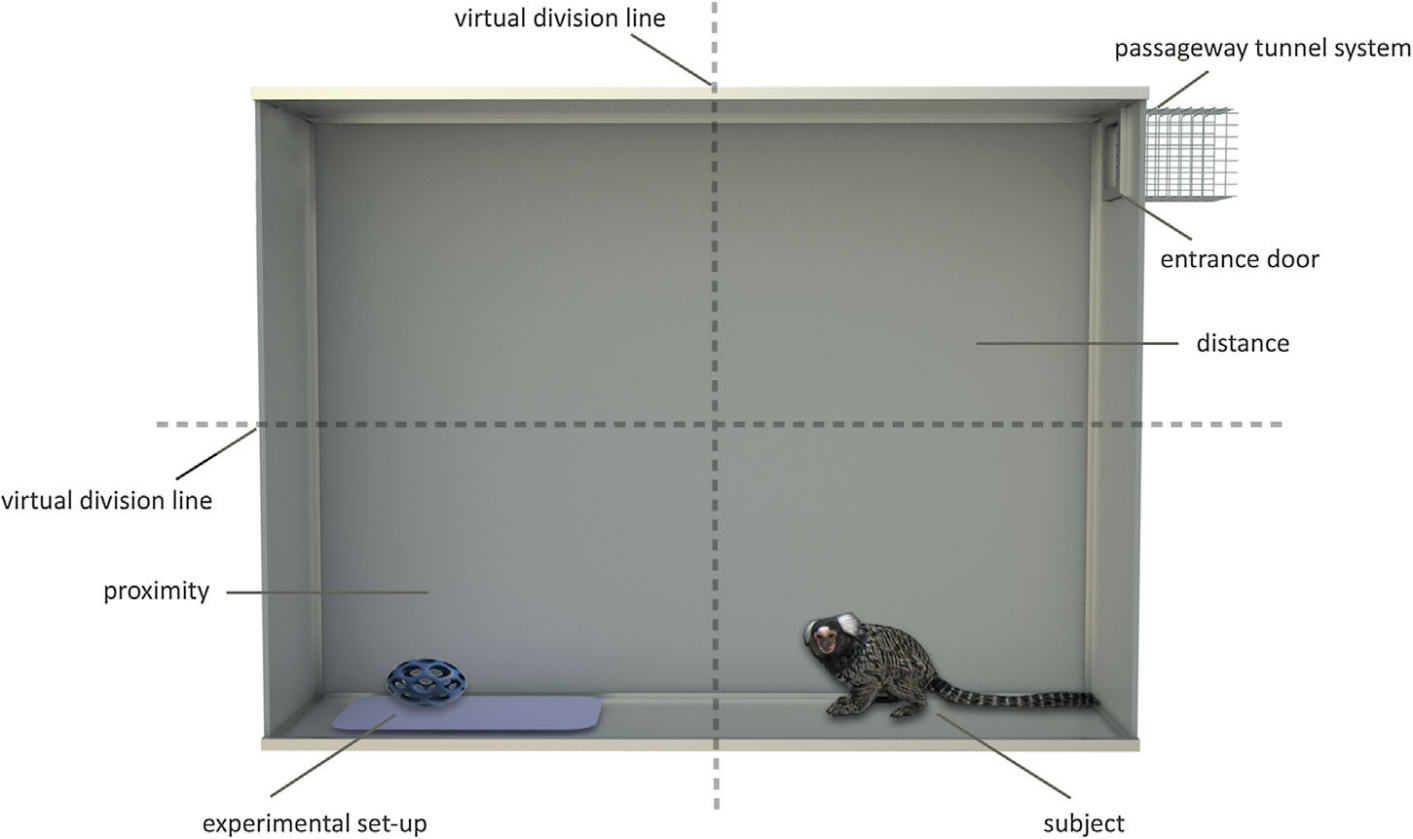
Frontal view of the experimental cage. Horizontal and vertical dashed lines represent virtual division of the experimental cage, i.e., into four different compartments.

Experimental testing occurred between February and May 2012. All experiments were conducted in an experimental cage (146 × 36 × 110 cm) (see Fig. 1). Before experiments began, the subjects received a 2‐week habituation phase with the experimental cage, the passageway system, the experimental routine and the experimenter (VŠ). During this time, the monkeys had access to the experimental cage for 30 min each day with food rewards, first as a whole family group and later individually.

Each experiment started when the entrance door of the experimental cage opened and lasted 5 min. The experimental set‐up was placed on an opaque plastic plate in the furthest point of the experimental cage (on the ground, diagonal to entrance door). The plate was changed for the different family groups to avoid olfactory interference. For the purpose of analysis, we virtually divided the experimental cage into four different compartments. Thus, the compartment containing the opaque plastic plate represented “proximity” (i.e., near to the experimental set‐up), whereas the one furthest away from it represented “distance” (i.e., far from the experimental set‐up) (see Fig. 1).

**Figure 2:**
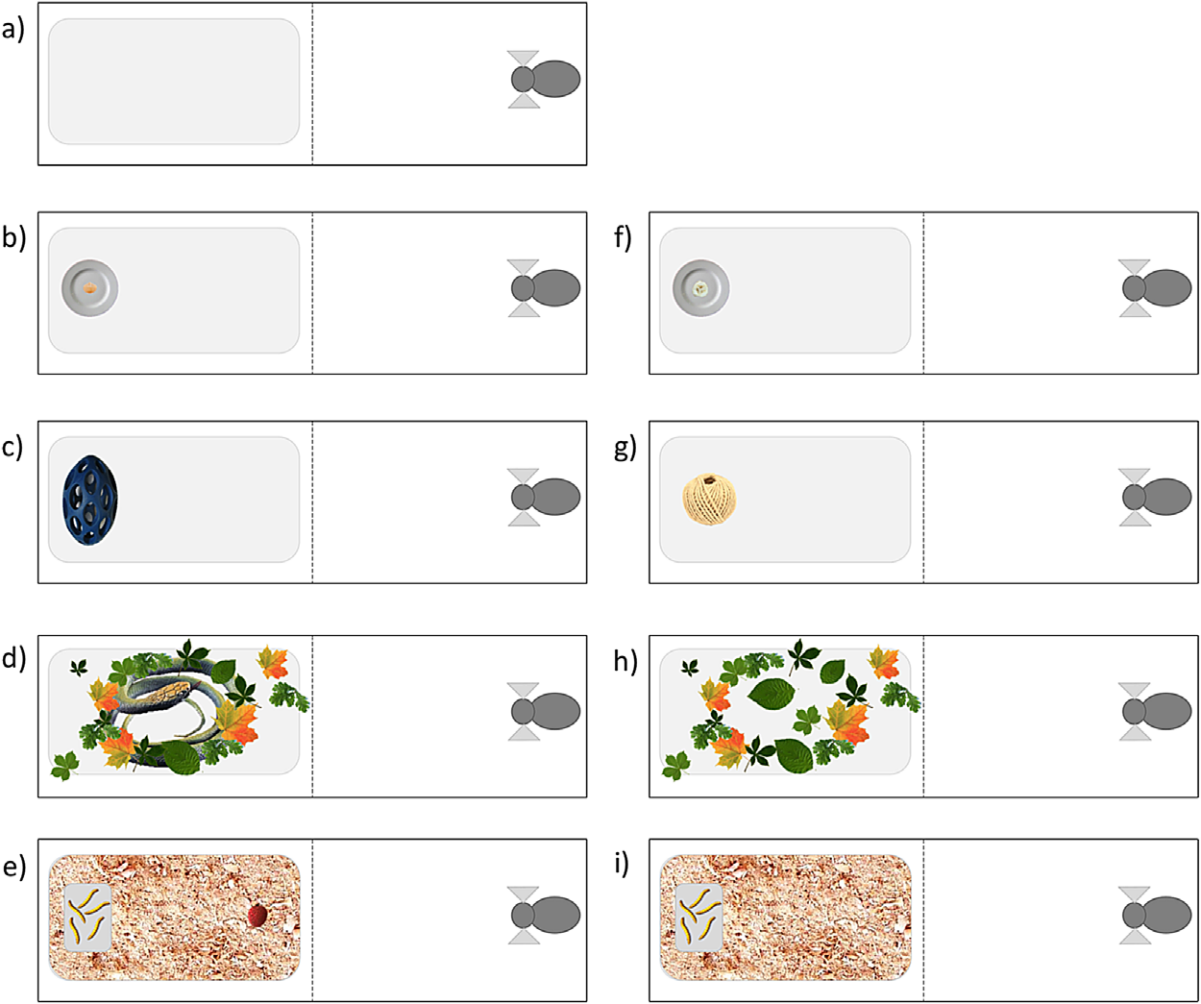
Bird's eye view of the test design: (**a**) GA; (**b**) tNF; (**c**) tNO; (**d**) tP; (**e**) tFUR; and control design: (**f**) cNF; (**g**) cNO; (**h**) cP; and (**i**) cFUR. The dashed line represents the virtual division of the experimental cage into the two bottom compartments.

Tests were conducted in the morning (9–12 am). We tested all animals in five different tests: (i) General Activity; (ii) Novel Food; (iii) Novel Object; (iv) Predator; and (v) Foraging Under Risk, and their controls: (vi) Novel Food Control; (vii) Novel Object Control; (viii) Predator Control; and (ix) Foraging Under Risk Control (Fig. 2). All subjects were tested with only one of the tests per testing day, with a 5 days break between testing days. Three days before the testing day, animals were tested with a matched control, to be able to isolate the effects of the testing from reactions to the testing situation, i.e., to be able to carefully interpret behavioral responses to novelty, predator, and other contexts as suggested by Carter [2013] (e.g., in the food related tasks controls were done to distinguish between food motivation and responses to novelty). All tests were conducted on two different occasions: the first test session was followed by a 14 days break without testing, and then the second test session was repeated. The order of subjects and tests was randomized, except for the General Activity Test (GA), which was always conducted first for all the monkeys.

### Tests

The GA measured the baseline behavior of the subjects in the empty experimental cage, which allowed us to specifically target the personality trait Activity (for a graphical representation of all tests and their controls see Fig. 2). The Novel Food Test (tNF) measured the behavior of the subjects confronted with a piece of novel food; i.e., we placed a novel food item (a macadamia nut in the first test session, a chestnut in the second test session) on a porcelain plate already known to the animals, in the experimental cage. Similarly, the Novel Object Test (tNO) measured the behavior of the subjects confronted with a novel object (a small green spiky plastic ball in the first test session, a big blue plastic ball with holes in the second test session). Both novelty tests were designed to target the personality trait Exploration‐Avoidance. The Predator Test (tP) measured the behavior of the subjects faced with a (model of a) predator (a plastic snake model placed on the opaque plastic plate and partially hidden in leaves). The Foraging Under Risk Test (tFUR) measured the behavior of the subjects confronted with a food reward and a potentially dangerous stimulus at the same time. In a pilot experiment, the subjects emitted mobbing/vigilance calls in the proximity of the skin of a lychee fruit. We assume that the texture resembles the skin texture of a predator, most likely a snake. Therefore, we used lychee fruit together with skin as a proxy for a dangerous stimulus. We covered the experimental plate with saw dust, placed a small transparent box containing valuable food rewards (five mealworms) on the furthermost part of the experimental plate, and placed the lychee fruit in front of the box. Both tests with “dangerous” stimuli were designed to target the personality trait Boldness‐Shyness.

### Controls

Experimental procedures of the controls were similar to their corresponding tests: in the Novel Food Control (cNF) we placed a familiar food item (a small piece of banana in both test sessions) on the porcelain plate instead of a novel food; in the Novel Object Control (cNO) a familiar object (string ball) instead of a novel object; in the Predator Control (cP) we did not hide a model of a predator in the leaves, but just placed the leaves on the experimental plate; and in the Foraging Under Risk Control (cFUR) no lychee fruit was placed in front of the transparent box containing the valuable food rewards.

### Data Coding

We recorded all behaviors of the subjects in the experimental cage from two different angles using two video cameras. One camera (SONY DCR‐SR35E) was placed on a tripod in front of the cage (focusing on the experimental set‐up), and the other camera (SANYO VPC‐WH1) was handled by VŠ, focusing on the subject and its behavior. We analyzed the videos using Solomon coder beta v. 12.09.02 [Péter, 2012]. For each test, we coded several behavioral parameters (see SEM, Table SI for more details on the variables).

For reliability purposes TG recoded 10% of the tests. Inter‐observer reliability was excellent both for frequencies (ICC (3,1) = 1.0, 95% CI lower, upper = 1.0, 1.0, *F* = 21866.712, *P* < 0.001) and durations and latencies (ICC (3,1) = 0.990, 95% CI lower, upper = 0.980, 0.995, *F* = 206.596, *P* < 0.001).

### Data Analysis

We analyzed the data using SPSS Statistics v. 20.0 (IBM). First, we tested for consistency over time. To estimate the repeatability of the behavioral measures from tests in the first and second test session, we used intra‐class correlation coefficients (ICCs). This coefficient is a mathematical equivalent to the standard repeatability test, i.e., it accounts for the proportion of variation in behavior that is responsible for inter‐individual variation, compared to that of intra‐individual variation [Falconer & Mackay, 1996; Lessells & Boag, 1987]. As personality is defined based on temporal consistency, the ICC value of the two repeatable variables had to show significant repeatability (*P* < 0.05) in order for a variable to be included in further analyses (see SEM, Table SII for significantly repeatable variables, and all variables measured). Subsequently, we calculated an individual mean value for these variables over the two repeated experiments.

Second, we tested the consistency of variables across different tests that we assumed were part of the same context (i.e., novelty (tNF and tNO), dangerous stimulus (tP and tFUR)) using ICCs, to identify cross‐contextual consistency of each behavioral variable. A variable was considered contextually consistent if the ICC value of the same variables from two different tests was significant (*P* < 0.05) (see SEM, Table SIII). If so, we calculated an individual mean value across the experiments. However, since the tests for contextual consistency were based on how we perceived contextual similarities, which might differ from the perception of the marmosets themselves, we also tested other contextually similar combinations (e.g., food‐related: tNF and tFUR, predator/neophobia‐related: tP, tFUR, tNO, and tNF). Also, we did not omit the variables that did not show contextual consistency, entering the measures of the different tests as separate variables into further analyses instead.

Third, we entered all remaining variables into a principal component analysis (PCA), to investigate whether and how these variables were associated with each other as traits. Eigenvalues (>1) and scree plots were used to assess the number of factors to extract. The PCA‐solution was Varimax‐rotated and variable loadings >±0.4 were considered salient (Table I). The analysis was repeated with a direct Oblimin rotation to elucidate the independence of the components [Tabachnick & Fidell, 2007]. Additionally, due to the relatively small sample size (*N *= 21), which could potentially lead to an unreliable solution in the PCA, we used a bootstrapping procedure to evaluate the stability of the factor structure [Diaconis & Efron, 1983; Zientek & Thompson, 2007]. A bootstrap component (or factor) analysis is useful for ascertaining the number of factors/components to retain or the replicability of the pattern/structure coefficients [cf. Lorenzo‐Seva & Ferrando, 2003; Zientek & Thompson, 2007]. In this procedure, separate principal component analyses were conducted on subsets of the sample (i.e., 1,000 random resamples) [cf. Capitanio, 1999], and we used a program syntax for SPSS published by Zientek & Thompson [2007] (see SEM, Table SIV). Furthermore, we used the regression method to obtain component scores for the obtained PCA constructs. This method produces scores that have a mean of zero and a variance equal to the squared multiple correlation between the estimated and the true component values [cf. Massen et al., 2013].

**Table I:** Variable Loadings in Principal Component Analysis (PCA)

	Component				
	Boldness‐Shyness in Foraging	Boldness‐Shyness in Predation	Stress‐Activity	Exploration‐Avoidance	Communalities
% of variance explained	38.9	18.23	13.61	10.4	
Eigenvalue	6.223	2.917	2.177	1.663	
Stress behavior, tNF		0.478	**0.804**		0.882
Self‐grooming, mean				**0.794**	0.660
Manipulation, tNF			−0.438	−0.616	0.774
Manipulation, tFUR	−**0.930**				0.934
Contact calls, tP		**0.910**			0.857
Contact calls, GA				**0.824**	0.751
Vigilance calls, tFUR	**0.784**				0.799
Vigilance calls, tP		−0.442			0.401
Body latency, tP		**−0.846**			0.838
Body latency, tFUR	**0.890**				0.905
Touch latency, tFUR	**0.934**				0.929
Touch latency, tNF			−0.493	0.689	0.836
Locomotion, mean			**0.800**		0.731
Compartment alternations, mean			**0.897**		0.928
Proximity, mean	**−0.852**	0.413			0.902
Distance, mean	0.632	−0.616			0.854

Varimax rotation with Kaiser normalization. Loadings >0.7 and <−0.7 are indicated in boldface. Communalities indicate a proportion of each variable's variance that can be explained by the principal components.

We used Generalized Linear Mixed Models (GLMMs) to assess the influence of age (continuous variable, 2–13 years), sex (12 males, 9 females), and family group (1, 2, and 3) on the derived component scores. In the initial full models, we included group, sex, age, and all two way interactions as fixed factors. Thereafter, we used a backward step‐wise approach to find the best fitting model based on comparisons of the corrected Akaike Information Criteria (cAIC). In the SEM, we report best fitting models (see SEM, Table SV). Based on the results of the models, we calculated post‐hoc analyses using Mann–Whitney U‐tests. For those post‐hoc analyses, we report *P*‐values after Holm Bonferroni correction [Holm, 1979]. Finally, we compared the temporally significantly repeatable behavioral variables from the tests with the same variables from the controls (see SEM, Table SVI) using Wilcoxon Signed Rank tests. All tests were two‐tailed and we set alpha to 0.05.

## RESULTS

We found that across the two test sessions, 24 variables were significantly repeatable (out of a total of 62 variables measured across different experiments) (see SEM, Table SII), indicating temporal consistency of these behaviors between the two test sessions. The ICC repeatability values ranged from 0.37 to 0.87 (see SEM, Table SII). Only these 24 behavioral variables were included in further analyses of cross‐contextual consistency. We first calculated an individual mean value of these variables over the two repeated experiments and then tested their internal consistency between different experiments (see SEM, Table SIII). We found that some of the variables showed significant cross‐experimental consistency (i.e., “locomotion” in GA, tNF, and tNO, ICC = 0.631, *P* = 0.004; “compartment alternations” in tNF and tP, ICC = 0.769, *P* < 0.001; “proximity” in tNO and tFUR, ICC = 0.655, *P *= 0.011; “distance” in tNO, tP, and tFUR, ICC = 0.694, *P* < 0.001; “self‐grooming” in tNF and tNO, ICC = 0.899, *P* < 0.001), whereas others did not (see SEM, Table SIII). The variables that showed significant consistency across experiments were averaged (i.e., the single mean value was calculated across different experiments), to obtain a single trait score for further analyses [cf. Massen et al., 2013]: “self‐grooming”, “locomotion”, “compartment alternations”, “proximity”, and “distance”. Cross‐experimentally inconsistent variables were kept for further analyses as unaveraged scores: “manipulation”, “contact calls”, “vigilance calls”, “body latency”, and “touch latency”. Similarly, “stress behavior” as the only temporal repeatable measure of its kind was also kept as a single variable and included as such into further analyses.

To investigate whether and how these variables (i.e., trait scores) are associated with each other as constructs, variables were entered in a PCA. In sum, 16 variables were entered into the PCA to assess the covariance among them. The PCA‐solution was Varimax‐rotated and variable loadings >±0.4 were considered salient (Table I). The analyses indicated appropriate sampling adequacy (Kaiser–Meyer–Olkin measure, KMO = 0.501; Bartlett's Test of Sphericity, *P* < 0.001), and all variables had communality estimates >0.401. We then evaluated the stability of the factor structure with running a bootstrapped PCA (i.e., 1,000 random resamples). We used a program syntax for SPSS, published by Zientek & Thompson [2007], with which we could examine standard errors, compare the sample to mean bootstrap results, and investigate the ratio of the mean bootstrap results to standard errors [Zientek & Thompson, 2007]. Indeed, our factor solution was remarkably stable (see SEM, Table SIV). We extracted four components, which together explained 81.13% of the variance. The first component explained 38.9% of the variance. This component had high positive loadings (>0.7) of “vigilance calls”, “body latency”, and “touch latency” in tFUR, and high negative loadings (<−0.7) of “manipulation” in tFUR and of the mean value of “proximity” in the different tests. This component also had salient positive loadings (>0.4) of the mean value of “distance” in the different tests. Thus, it consisted of variables related to Boldness‐Shyness and Exploration‐Avoidance tendencies. However, as the majority of variables that loaded on this component were related to tFUR, we labeled it Boldness‐Shyness in Foraging. The second component explained 18.23% of the variance and had high positive loadings (>0.7) of “contact calls” in tP, and high negative loadings (<−0.7) of “body latency” in tP. This component also had salient positive loadings (>0.4) of “stress behavior” in tNF and of the mean value of “proximity” in the different tests (albeit weaker than in the first component), and salient negative loadings (<−0.4) of “vigilance calls” in tP and of the mean value of “distance” in the different tests. Thus, this component consisted of variables related to Boldness‐Shyness tendencies. However, as the variables were mainly related to the predatory context, we labeled this component Boldness‐Shyness in Predation. The third component explained 13.61% of the variance. It had high loadings (>0.7) of the mean values of “locomotion” and “compartment alternations” in the different tests and of “stress behavior” in tNF. Moreover, it had salient negative loadings (<−0.4) of “manipulation” and of “touch latency” in tNF. Since variables that had highest factor loadings in this component were related to stress and activity [see SEM, Table SI, Barros et al., 2000; Stevenson & Poole, 1976] we labeled this component Stress‐Activity. Finally, the fourth component explained 10.4% of the variance. It had high positive loadings (>0.7) of the mean value of “self‐grooming” in the novelty tests (tNF & tNO) and of “contact calls” in GA. It also had salient positive loadings (>0.4) of “touch latency” in tNF, and salient negative loadings (<−0.4) of “manipulation” in tNF. As it consisted of variables related mainly to exploration tendencies, we labeled it Exploration‐Avoidance. We re‐ran the analysis with a direct Oblimin rotation and this rotation resulted in a rotated solution almost identical to the Varimax‐rotated one regarding the variable loadings. Moreover, the extracted components did not correlate strongly with each other (highest factor intercorrelation after direct Oblimin rotation: *r* = −0.24).

Finally, we ran Generalized Linear Mixed Models (GLMMs) to assess the effect of sex, age, and group on all four factors (components). The best fitting models revealed no sex or age differences (see SEM, Table SV). We did find a difference between groups with regard to Exploration‐Avoidance (*F* = 26.544, df 1,2 = 2, 15, *P *< 0.001), but not for any other factor. Additionally, we found an interaction‐effect of group and sex, also with regard to Exploration‐Avoidance (*F* = 14.996, df 1,2 = 3, 15, *P* < 0.001), but not for any other factors. In contrast, all other interactions were either not present in the final models or non‐significant irrespective of the factor tested (see SEM, Table SV).

**Figure 3:**
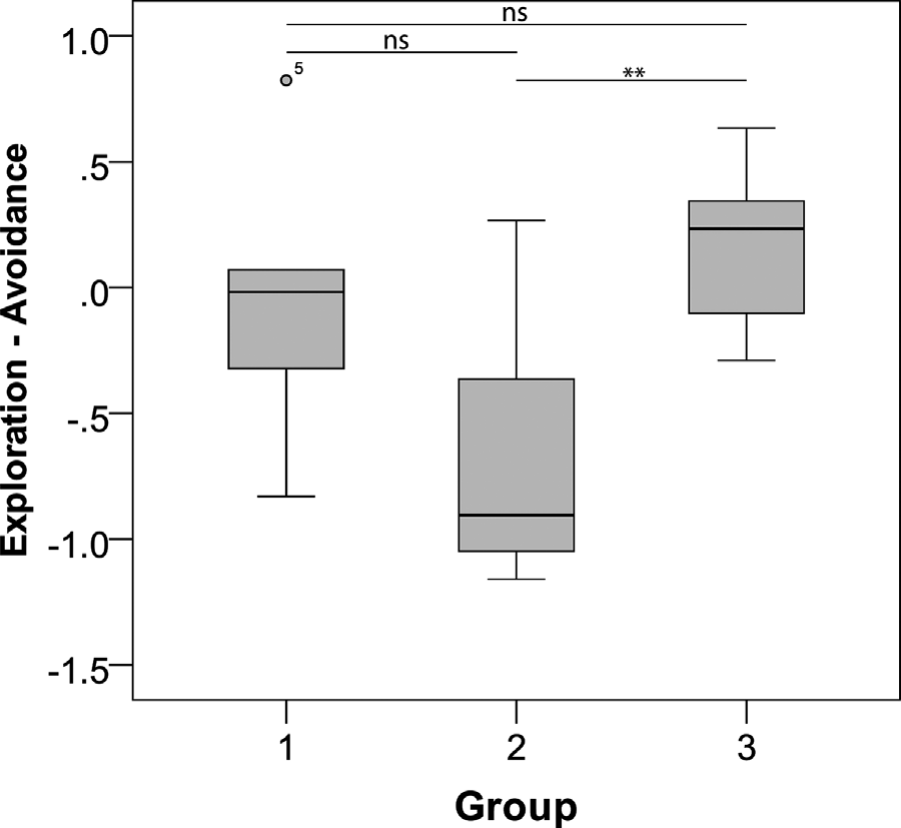
Exploration‐Avoidance factor scores per group; box limits indicate the 25th and 75th percentiles as determined by SPSS software; whiskers extend 1.5 times the interquartile range from the 25th and 75th percentiles, outliers are represented by dots. *N* = 5, 7, 8 sample points. **P < 0.01, ns = non‐significant.

Visual inspection of our data revealed that the interaction effect of group and sex on Exploration‐Avoidance might have been solely due to one female, as her factor score was almost two standard deviations higher than the rest of her group. Re‐analyses of the data without this female confirmed this, since the interaction effect was lost in the subsequent final model on Exploration‐Avoidance (group*sex; *F* = 0.141, df 1,2 = 2,15, *P* = 0.870). In contrast, the initial group effect remained significant (*F* = 5.248, df 1,2 = 2, 15, *P* = 0.019), suggesting it was a consistent effect. Post‐hoc analyses without the female revealed that group members of group 2 had significantly lower factor scores (after Holm–Bonferroni correction) with regard to Exploration‐Avoidance than members of group 3, whereas all other combinations of groups showed no significant differences (Mann–Whitney U‐tests: group 1 vs. group 2: *U* = 7, *Z* = −1.705, *P* = 0.106, group 2 vs. group 3: *U* = 5, *Z* = −2.662, *P* = 0.006, group 1 vs. group 3: *U* = 14, *Z* = −0.878, *P* = 0.380; Fig. 3).

Finally, we compared tests with the controls using Wilcoxon Signed Rank tests. As expected, behavioral responses always differed significantly between the tests and the corresponding controls in predator and foraging under risk conditions, and they differed significantly in most of the food and object conditions (see SEM, Table SVI). Consequently, these results validated our experimental approach.

## DISCUSSION

In this study, we investigated the consistency of inter‐individual differences in common marmosets, with the aim to show three non‐social personality traits (Activity, Boldness‐Shyness, and Exploration‐Avoidance). In contrast to previous studies, we tested the monkeys in a solitary setting, using five different experiments (GA, tNF, tNO, tFUR, tP) and their corresponding controls (cNF, cNO, cFUR, cP). Repeated solitary testing allowed us to eliminate possible social influences such as audience effects and/or social facilitation, and to obtain unbiased individual personality scores at two different time points. The use of controls allowed us to isolate the effects of testing from reactions in the test situation. We found that the individuals differed in most of their behavior consistently over time and across different contexts, which perfectly fits the definition of personality [Réale et al., 2010]. These repeatable behaviors formed a construct of four major dimensions: Boldness‐Shyness in Foraging, Boldness‐Shyness in Predation, Stress‐Activity, and Exploration‐Avoidance. We found no sex or age differences in these components, but we detected a difference between the groups with regard to the component Exploration‐Avoidance.

We found temporal repeatability in 24 (out of 62) behavioral variables and the degree of this repeatability was within the repeatability range of behaviors described in other species, as it has been argued that approximately 35% of the variation among individuals in behavior can be attributed to personality [Bell et al., 2009]. Moreover, we did not only consider significance, but also effect‐sizes (i.e., ICC‐values), which were moderate to very high. Note that behavioral responses varied in their temporal consistency in different experiments: i.e., whereas many behaviors (e.g., target manipulation, calls, movement patterns, position in the cage, some latencies) were fairly repeatable, others, like entering latencies, were not. This finding is to some extent in accordance with previous studies. Massen and colleagues [2013] suggested that this temporal inconsistency in latencies could be due to a habituation effect, specifically in novelty (here, tNF or tNO), or a decrease of the perceived threat (here, tP or tFUR) with regard to predator models.

We tested 24 mean values of temporally consistent variables for their contextual consistency, across multiple tests. We predicted that the monkeys would have a similar response in tests of the same personality trait (Activity: GA, Boldness‐Shyness: tFUR and tP, Exploration‐Avoidance: tNO and tNF) [Stamps & Groothuis, 2010], or in tests of other contextually similar combinations (e.g., food‐related: tNF and tFUR, predator/neophobia‐related: tP, tFUR, tNO, and tNF). As expected, some variables did indeed show significant cross‐experimental consistency. For example, locomotion was consistent in GA, tNF, and tNO, suggesting that novelty might have an impact on the excitement, and therefore on the duration of locomotion, as this was consistent not only in tNF and tNO, but also in GA which was conducted first for all monkeys. Self‐grooming was consistent in novelty tests (tNF and tNO). Time spent in proximity of the stimulus was consistent across some novelty and food‐related tests (tNO and tFUR) which might be explained by a high level of curiosity. Time spent distant from the stimulus was consistent in two contexts with frightening stimuli (tP and tFUR) and one context with novelty (tNO). The number of compartment alternations (potential measure of Activity) was consistent in one novelty (tNF) and one predatory context (tP), which might be explained by both predator‐avoidance mechanisms and neophobia. Note that there could be several reasons why not all of our parameters were contextually consistent. For instance, latency to approach could be affected by different motivations in a predator context and in a novel food context. Even though the definition of personality does not require consistency in both time and context, we are open to the argument that a “personality” trait that is not consistent across contexts might actually reflect independent traits.

The PCA analysis indicated four independent principal components (Table I), and the factor solution remained stable after performing a bootstrapping procedure of the PCA (see SEM, Table SIV). Although this statistical tool supports the robustness of our results, we have to interpret our findings with caution due to the relatively small sample size. Notably, we did not expect that Boldness‐Shyness would form two separate components, based on the context in which it was tested. The first principal component consisted mostly of risk‐taking variables found in the foraging context. Variable loadings of this component indicated that shyer individuals emitted more vigilant calls and took longer to approach the stimulus, while bolder ones stayed in proximity to the stimuli and manipulated the food reward in tFUR for longer periods of time. Vigilant responses to threatening stimuli have already been used to classify boldness in male fowl alarm calls in response to a simulated overhead predator [cf. Carter et al., 2012; Nelson et al., 2008]. The second principal component consisted of similar behaviors, but were predominantly found in a predator context. In other words, vigilance calls loading on this component were mostly emitted by shy individuals that took longer to approach the predator model in tP and spent most time further away from the stimuli. In contrast, bolder individuals spent more time close to the stimuli, showed more stress behaviors and emitted more contact calls. In a study on vervet monkeys (*Cercopithecus aethiops sabaeus*), the boldest males also placed themselves at the highest risk of injury while responding to an intruder, whereas shy individuals took a safer, less risky approach [Fairbanks, 2001]. Similarly, Coleman & Wilson [1998] found that bolder sunfish engage more in predator inspection than shy individuals. Furthermore, they also fed more when they were exposed to a novel environment and acclimated more quickly to the laboratory setting than shy individuals. Interestingly, none of these studies found Boldness‐Shyness forming two separate components. Further studies may reveal whether our findings can be treated as a special outcome of our tests/analyses or whether individuals consistently express different traits according to the type of risk involved. If the latter is true, such traits may have implications for the monkeys’ life‐histories.

Since most of the behaviors that loaded on the third principal component were related to activity of subjects and their stress response in given tests, we labeled this component Stress‐Activity. Activity is often found as an independent personality trait, both in primates [Koski, 2011; Schneider et al., 1991; Stevenson‐Hinde & Zunz, 1978] and non‐primates [Bell, 2005], and stress responses (sometimes labeled as “Excitability”) have been shown as independent personality trait also in other studies [Capitanio, 1999]. Increased locomotion is one of the stress indicators in common marmosets [Bassett et al., 2003], and sometimes variables related to subjects’ activity and to their stress responses load on the same component (e.g., “Excitable”) [Stevenson‐Hinde et al., 1980b], so it is not surprising that we found the same pattern in this study. Finally, the fourth component labeled Exploration‐Avoidance consisted of traits related to explorative tendencies of marmosets. In other words, more explorative individuals manipulated objects longer and were faster to approach novel food, whereas avoidant individuals elicited more contact calls and showed more self‐grooming. Similar to the study by Massen and colleagues [2013] on chimpanzees, Exploration‐Avoidance formed a separate construct from Boldness‐Shyness, supporting the notion that neophobia and boldness might be independent constructs [Carter et al., 2012; Greenberg & Mettke‐Hofmann, 2001].

We found no sex or age differences within the components, suggesting that our personality components are unaffected by demographic effects. Similar results were obtained in other studies on marmosets [Kemp & Kaplan, 2011; Koski & Burkart, 2015; Rogers, 1999], and barnacle geese [Kurvers et al., 2009]; in contrast, studies on zebra finches [Schuett & Dall, 2009], gray mouse lemurs [Dammhahn, 2012], vervet monkeys [McGuire et al., 1994], and chimpanzees [Massen et al., 2013] did find effects of sex and/or age. Interestingly, our results did reveal differences between groups; i.e., with regard to Exploration‐Avoidance, and an interaction‐effect of group and sex with regard to Exploration‐Avoidance. Members of group 2 had significantly lower factor scores in Exploration‐Avoidance than members of group 3. Even though there was considerable within‐group variation of this factor score (see error bars in Fig. 3), it seems that the personality traits of members from the same family group were more similar to each other than to members of a different group. These differences cannot be explained solely by genetic differences as the monkeys of both groups are not only genetically related to members of their own group, but also to members of the other group [cf. Koski & Burkart, 2015]. It should be noted that the results of these regression analyses have to be taken with caution, as the subject to variable ratio in the GLMMs was not very strong; i.e., 3.5:1 (but see Austin & Steyerberg, 2015, that report that a subject to variable ratio of 2:1 is sufficient for an adequate estimation of regression coefficients, standard errors, and confidence intervals).

The found group differences support the notion that social environment can have a large influence on the behavior of individuals [Kralj‐Fišer et al., 2007; Sih & Bell, 2008]. Namely, it can both restrict the expression of behavioral traits through conformity and enhance them through facilitation [Webster & Ward, 2011], making the behavior of individuals of the same group more similar. In a study on 75 chimpanzees in a social setting, significant group‐level differences were found in four social personality traits that could not be explained by ecological factors [Koski, 2011]. Although we tested individuals in a solitary setting, we nevertheless obtained a group‐specific expression of an Exploration‐Avoidance personality trait, and to our knowledge, this is the first study to show such a result using repeated individual testing in common marmosets. All our groups consist of an unrelated male and female and their offspring, so one possible explanation might be that similarity within groups is due to a combination of shared genetics and shared early social environment, which might be particularly true for offspring reared in these groups [cf. Fairbanks, 2001; Schneider et al., 1991; Suomi, 1987]. However, as group 2 and group 3 are genetically related, these differences cannot be solely explained by shared genetics. The other plausible explanation might be group‐level similarity in personality (i.e., “group personality” [Koski & Burkart, 2015]), even outside of an immediate social context. This behavior might be especially important for group‐living species that might benefit from grouping when faced with predators [Landeau & Terborgh, 1986]. Callitrichids are no exception to this rule, with a wide array of anti‐predator strategies [Caine, 1993; Ferrari & Ferrari, 1990]. When foraging for prey, resting, socializing, playing, grooming, etc., it seems to be of utmost importance for this species to maintain social cohesion within their family group [Fragaszy & Visalberghi 2004; Stevenson & Rylands, 1988]. Indeed, it has been shown that common marmosets’ foraging behavior (that could be associated with exploratory behavior) is influenced by social learning both in captive [Bugnyar & Huber, 1997; Voelkl & Huber, 2000] and in wild populations [Gunhold et al., 2014a,2014b], and thus group‐level similarity in personality with regard to Exploration‐Avoidance may be beneficial.

In a recent study by Koski & Burkart [2015], common marmosets were found to show social modification of their personality traits across social and solitary conditions in a battery of tests. Moreover, in both conditions individuals showed group‐level similarity in Boldness‐Shyness. However, the same finding was not retained in Exploration‐Avoidance, where marmosets adhered to their group only in a social context. The authors hypothesized that the mechanism that influences exploratory behavior might be influenced by group members, thus leading to social facilitation. Our study, in contrast, did find a group effect on Exploration‐Avoidance in a solitary setting, suggesting that this group effect is not the result of short‐term social facilitation, but rather of a long‐term process that produces group‐level similarity in behavior. As our study applied a more thorough approach with regard to personality in the solitary setting, we would like to suggest that marmosets may also show group‐level similarity in Exploration‐Avoidance in a solitary setting. This adds an important additional piece of knowledge about group‐level similarity in personality, providing a stronger argument for the possible presence of not only short‐term effects, but also long‐term social effects leading to group cohesion, and possibly increasing group coordination and cooperation [Koski & Burkart, 2015]. Interestingly, unlike the Koski & Burkart [2015] study, our study did not find a group difference in Boldness‐Shyness scores. It may be that Boldness‐Shyness is indeed less susceptible to the effects of the social environment and is regulated by more internal genetic mechanisms. As our groups 2 and 3 were genetically related, this might be a plausible explanation for the absence of “group personality” in this trait, as opposed to the study by Koski & Burkart [2015], where the monkeys did not share the same genetic background. However, this remains to be further investigated.

In sum, we found consistent inter‐individual differences in 21 common marmosets in a solitary setting, using five different experiments (GA, tNF, tNO, tFUR, tP) and their corresponding controls (cNF, cNO, cP, cFUR). Individuals behaved consistently over time and across different contexts, revealing four major personality dimensions: Boldness‐Shyness in Foraging, Boldness‐Shyness in Predation, Stress‐Activity, and Exploration‐Avoidance. To our knowledge, this is the first study in which Boldness‐Shyness appeared as two separate components, which calls for further investigation. A significant group difference with regard to the Exploration‐Avoidance component in our solitary setting suggests that members of the same family group had more similar personalities than members of a different group in at least one trait, which is in line with the idea of group‐level similarity in personality.

## Supporting information

Additional supporting information may be found in the online version of this article at the publisher's web‐site.

Supporting Information.

## References

[ajp22566-bib-0001] Austin PC , Steyerberg EW . 2015 The number of subjects per variable required in linear regression analyses. Journal of Clinical Epidemiology 68:627–636. 25704724 10.1016/j.jclinepi.2014.12.014

[ajp22566-bib-0002] Barros M , Boere V , Huston JP , Tomaz C . 2000 Measuring fear and anxiety in the marmoset (*Callithrix penicillata*) with a novel predator confrontation model: effects of diazepam. Behavioural Brain Research 108:205–211. 10701664 10.1016/s0166-4328(99)00153-9

[ajp22566-bib-0003] Bassett L , Buchanan‐Smith HM , McKinley J , Smith TE . 2003 Effects of training on stress‐related behavior of the common marmoset (*Callithrix jacchus*) in relation to coping with routine husbandry procedures. Journal of Applied Animal Welfare Science 6:221–233. 14612270 10.1207/S15327604JAWS0603_07

[ajp22566-bib-0004] Bell AM , Hankison SJ , Laskowski KL . 2009 The repeatability of behaviour: a meta‐analysis. Animal Behaviour 77:771–783. 24707058 10.1016/j.anbehav.2008.12.022 PMC3972767

[ajp22566-bib-0005] Bell AM . 2005 Behavioural differences between individuals and two populations of stickleback (*Gasterosteus aculeatus*). Journal of Evolutionary Biology 18:464–473. 15715852 10.1111/j.1420-9101.2004.00817.x

[ajp22566-bib-0006] Bergmüller R , Taborsky M . 2010 Animal personality due to social niche specialisation. Trends in Ecology and Evolution 25:504–511. 20638151 10.1016/j.tree.2010.06.012

[ajp22566-bib-0007] Bouchard TJ , Loehlin JC . 2001 Genes, evolution, and personality. Behavior Genetics 31:243–273. 11699599 10.1023/a:1012294324713

[ajp22566-bib-0008] Bugnyar T , Huber L . 1997 Push or pull: an experimental study on imitation in marmosets. Animal Behaviour 54:817–831. 9344436 10.1006/anbe.1996.0497

[ajp22566-bib-0009] Caine NG . 1993 Flexibility and co‐operation as unifying themes in Saguin us social organisation and behaviour: the role of predation pressures In: Rylands A, editor. Marmosets and tamarins: Systematic, behaviour and ecology. Oxford, UK: Oxford University Press p 200–219.

[ajp22566-bib-0010] Capitanio JP . 1999 Personality dimensions in adult male rhesus macaques: prediction of behaviors across time and situation. American Journal of Primatology 47:299–320. 10206208 10.1002/(SICI)1098-2345(1999)47:4<299::AID-AJP3>3.0.CO;2-P

[ajp22566-bib-0011] Capitanio JP . 2004 Personality factors between and within species In: Thierry B, Singh M, Kaumanns W, editors. Macaque societies: A model for the study of social organisation. Cambridge UK: Cambridge University Press p 13–37.

[ajp22566-bib-0012] Capitanio JP , Widaman KF . 2005 Confirmatory factor analysis of personality structure in adult male rhesus monkeys (*Macaca mulatta*). American Journal of Primatology 65:289–294. 15772988 10.1002/ajp.20116

[ajp22566-bib-0013] Capitanio JP , Del Rosso LA , Calonder LA , Blozis SA , Penedo MCT . 2012 Behavioral effects of prenatal ketamine exposure in rhesus macaques are dependent on MAOA genotype. Experimental and Clinical Psychopharmacology 20:173–180. 22250657 10.1037/a0026773 PMC3481859

[ajp22566-bib-0014] Carere C , Maestripieri D . 2013 Introduction: animal personalities: who cares and why? In: Carere C, Maestripieri D, editors. Animal personalities: Behavior, physiology, and evolution. Chicago, IL: University of Chicago Press p 1–9.

[ajp22566-bib-0015] Carter AJ , Marshall HH , Heinsohn R , Cowlishaw G . 2012 How not to measure boldness: novel object and antipredator responses are not the same in wild baboons. Animal Behaviour 84:603–609.

[ajp22566-bib-0016] Carter AJ . 2013 On validity and controls in animal personality research: a comment on Galhardo et al. (2012). Biology Letters 9:20121080. 23676652 10.1098/rsbl.2012.1080 PMC3730612

[ajp22566-bib-0017] Carter AJ , Feeney WE , Marshall HH , Cowlishaw G , Heinsohn R . 2013 Animal personality: what are behavioural ecologists measuring? Biological Reviews 88:465–475. 23253069 10.1111/brv.12007

[ajp22566-bib-0018] Chapais B . 2001 Primate nepotism: what is the explanatory value of kin selection? International Journal of Primatology 22:203–229.

[ajp22566-bib-0019] Coleman K . 2012 Individual differences in temperament and behavioral management practices for nonhuman primates. Applied Animal Behaviour Science 138:106–113. 22518067 10.1016/j.applanim.2011.08.002 PMC3327443

[ajp22566-bib-0020] Coleman K , Wilson D . 1998 Shyness and boldness in pumpkinseed sunfish: individual differences are context‐specific. Animal Behaviour 56:927–936. 9790704 10.1006/anbe.1998.0852

[ajp22566-bib-0021] Costa PT , McCrae RR . 1992 Revised NEO Personality Inventory (NEO‐PI‐R) and NEO Five‐Factor Inventory (NEO‐FFI) manual. Odessa, FL: Psychological Assessment Resources p 101.

[ajp22566-bib-0022] Crockford C , Wittig RM , Seyfarth RM , Cheney DL . 2007 Baboons eavesdrop to deduce mating opportunities. Animal Behaviour 73:885–890.

[ajp22566-bib-0023] Croft DP , Krause J , James R . 2004 Social networks in the guppy (*Poecilia reticulata*). Proceedings of the Royal Society of London B 271(Suppl 6):S516–S519. 10.1098/rsbl.2004.0206 PMC1810091 15801620

[ajp22566-bib-0024] Dall SRX , Griffith SC . 2014 An empiricist guide to animal personality variation in ecology and evolution. Frontiers in Ecology and Evolution 2:1–7.

[ajp22566-bib-0025] Dammhahn M . 2012 Are personality differences in a small iteroparous mammal maintained by a life‐history trade‐off? Proceedings of the Royal Society B 13:2645–2651. 22398164 10.1098/rspb.2012.0212 PMC3350711

[ajp22566-bib-0026] Diaconis P , Efron B . 1983 Computer‐intensive methods in statistics. Scientific American 248:116–130.

[ajp22566-bib-0027] Digman JM . 1990 Personality structure: emergence of the Five‐Factor Model. Annual Review of Psychology 41:417–440.

[ajp22566-bib-0028] Dingemanse NJ , Both C , Drent PJ , van Oers K , van Noordwijk AJ . 2002 Repeatability and heritability of exploratory behaviour in great tits from the wild. Animal Behaviour 64:929–937.

[ajp22566-bib-0029] Fairbanks LA . 2001 Individual differences in response to a stranger: social impulsivity as a dimension of temperament in vervet monkeys (*Cercopithecus aethiops sabaeus*). Journal of Comparative Psychology 115:22–28. 11334215 10.1037/0735-7036.115.1.22

[ajp22566-bib-0030] Falconer DS , Mackay TFC . 1996 Introduction to quantitative genetics. 4th edition Harlow, Essex, UK: Longmans Green p 480.

[ajp22566-bib-0031] Ferrari SF , Ferrari MAL . 1990 Predator avoidance behaviour in the buffy‐headed marmoset, *Callithrix flaviceps* . Primates 31:323–338.

[ajp22566-bib-0032] Fragaszy D , Visalberghi E . 2004 Socially biased learning in monkeys. Learning and Behavior 32:24–35. 15161138 10.3758/bf03196004

[ajp22566-bib-0033] Freeman H , Gosling SD , Schapiro SJ . 2011 Methods for assessing personality in non‐human primates In: Weiss A, King J, Murray L, editors. Personality and behavioral syndromes in nonhuman primates. New York: Springer.

[ajp22566-bib-0034] Galton F . 1883 Inquiries into human faculty and its development. London: Macmillan.

[ajp22566-bib-0035] Gosling SD . 2001 From mice to men: what can we learn about personality from animal research? Psychological Bulletin 127:45–86. 11271756 10.1037/0033-2909.127.1.45

[ajp22566-bib-0036] Greenberg R , Mettke‐Hofmann C . 2001 Ecological aspects of neophobia and neophilia in birds. Current Ornithology 16:119–178.

[ajp22566-bib-0037] Grzimek B . 2003 Family: new world monkeys II In: Hutchins M, Kleiman D, Geist V, McDade M, editors. Grzimek's animal life encylopedia, Volume 14, Mammals III. 2nd Edition Farmington Hills, Michigan, USA: Gale Group p 115–133.

[ajp22566-bib-0038] Gunhold T , Whiten A , Bugnyar T . 2014a Video demonstrations seed alternative problem solving techniques in wild common marmosets. Biology Letters 10:20140439. 25187646 10.1098/rsbl.2014.0439 PMC4190960

[ajp22566-bib-0039] Gunhold T , Massen JJM , Schiel N , Souto A , Bugnyar T . 2014b Memory, transmission and persistence of alternative foraging techniques in wild common marmosets. Animal Behaviour 91:79–91. 24910466 10.1016/j.anbehav.2014.02.023 PMC4045399

[ajp22566-bib-0040] Gunhold T , Range F , Huber L , Bugnyar T . 2015 Long‐term fidelity of foraging techniques in common marmosets (*Callithrix jacchus*). American Journal of Primatology 77:264–270. 25231356 10.1002/ajp.22342 PMC4371577

[ajp22566-bib-0041] Hebb DO . 1946 Emotion in Man and Animal: an analysis of the intuitive processes of recognition. Psychological Review 53:88–106. 21023321 10.1037/h0063033

[ajp22566-bib-0042] Holm S . 1979 A simple sequentially rejective multiple test procedure. Scandinavian Journal of Statistics 6:65–70.

[ajp22566-bib-0043] Huntingford FA . 1976 The relationship between anti‐predator behaviour and aggression among conspecifics in the three‐spined stickleback, Gasterosteus Aculeatus. Animal Behaviour 24:245–260.

[ajp22566-bib-0044] Kemp C , Kaplan G . 2011 Individual modulation of anti‐predator responses in common marmosets. International Journal of Comparative Psychology 24:112–136.

[ajp22566-bib-0045] King JE , Figueredo AJ . 1997 The five‐factor model plus dominance in chimpanzee personality. Journal of Research in Personality 31:257–271.

[ajp22566-bib-0046] Koolhaas JM , de Boer SF , Coppens CM , Buwalda B . 2010 Neuroendocrinology of coping styles: towards understanding the biology of individual variation. Frontiers in Neuroendocrinology 31:307–321. 20382177 10.1016/j.yfrne.2010.04.001

[ajp22566-bib-0047] Koski SE , Burkart JM . 2015 Common marmosets show social plasticity and group‐level similarity in personality. Scientific reports 5:8878. 25743581 10.1038/srep08878 PMC5155412

[ajp22566-bib-0048] Koski SE . 2011 Social personality traits in chimpanzees: temporal stability and structure of behaviourally assessed personality traits in three captive populations. Behavioral Ecology and Sociobiology 65:2161–2174.

[ajp22566-bib-0049] Koski SE . 2014 Broader horizons for animal personality research. Frontiers in Ecology and Evolution 2:1–17.

[ajp22566-bib-0050] Kralj‐Fišer S , Scheiber IBR , Blejec A , Möstl E , Kotrschal K . 2007 Individualities in a flock of free‐roaming greylag geese: behavioral and physiological consistency over time and across situations. Hormones and Behavior 51:239–248. 17196200 10.1016/j.yhbeh.2006.10.006

[ajp22566-bib-0051] Krause J , James R , Croft DP . 2010 Personality in the context of social networks. Philosophical Transactions of the Royal Society of London B 365:4099–4106. 10.1098/rstb.2010.0216 PMC2992749 21078661

[ajp22566-bib-0052] Kurvers RHJM , Eijkelenkamp B , van Oers K , et al. 2009 Personality differences explain leadership in barnacle geese. Animal Behaviour 78:447–453.

[ajp22566-bib-0053] Landeau L , Terborgh J . 1986 Oddity and the “confusion effect” in predation. Animal Behaviour 34:1372–1380.

[ajp22566-bib-0054] Lessells CM , Boag PT . 1987 Unrepeatable repeatabilities: a common mistake. Auk 104:116–121.

[ajp22566-bib-0055] Lorenzo‐Seva U , Ferrando PJ . 2003 IMINCE: an unrestricted factor‐analysis‐based program for assessing measurement invariance. Behavior Research Methods 35:318–321. 10.3758/bf03202558 12834090

[ajp22566-bib-0056] Massen JJM , Koski SE . 2014 Chimps of a feather sit together: chimpanzee friendships are based on homophily in personality. Evolution and Human Behavior 35:1–8.

[ajp22566-bib-0057] Massen JJM , Sterck EHM , de Vos H . 2010 A review of close social associations in animals and humans: functions and mechanisms of friendship. Behaviour 147:1379–1412.

[ajp22566-bib-0058] Massen JJM , Antonides A , Arnold AMK , Bionda T , Koski SE . 2013 A behavioral view on chimpanzee personality: exploration tendency, persistence, boldness, and tool‐orientation measured with group experiments. American Journal of Primatology 75:947–958. 23649750 10.1002/ajp.22159

[ajp22566-bib-0059] McGuire MT , Raleigh MJ , Pollack DB . 1994 Personality features in vervet monkeys: the effects of sex, age, social status, and group composition. American Journal of Primatology 33:1–13. 10.1002/ajp.1350330102 31936927

[ajp22566-bib-0060] Nelson XJ , Wilson DR , Evans CS . 2008 Behavioral syndromes in stable social groups: an artifact of external constraints? Ethology 114:1154–1165.

[ajp22566-bib-0061] Nettle D , Penke L . 2010 Personality: bridging the literatures from human psychology and behavioural ecology. Philosophical Transactions of the Royal Society of London B 365:4043–4050. 10.1098/rstb.2010.0061 PMC2992738 21078656

[ajp22566-bib-0062] Péter A . 2012 Solomon Coder (version beta 12.09.02): A simple solution for behavior coding. http://solomoncoder.com/

[ajp22566-bib-0063] Réale D , Reader SM , Sol D , McDougall PT , Dingemanse NJ . 2007 Integrating animal temperament within ecology and evolution. Biological Reviews 82:291–318. 17437562 10.1111/j.1469-185X.2007.00010.x

[ajp22566-bib-0064] Réale D , Dingemanse NJ , Kazem AJN , Wright J . 2010 Evolutionary and ecological approaches to the study of personality. Philosophical Transactions of the Royal Society of London B 365:3937–3946. 10.1098/rstb.2010.0222 PMC2992753 21078646

[ajp22566-bib-0065] Rogers LJ . 1999 Factors associated with exploration in marmosets: age, gender and hand preference. International Journal of Comparative Psychology 12:93–109.

[ajp22566-bib-0066] Rouff JH , Sussman RW , Strube MJ . 2005 Personality traits in captive lion‐tailed macaques (*Macaca silenus*). American Journal of Primatology 67:177–198. 16229004 10.1002/ajp.20176

[ajp22566-bib-0067] Schneider ML , Moore CF , Suomi SJ , Champoux M . 1991 Laboratory assessment of temperament and environmental enrichment in rhesus monkey infants (*Macaca mulatta*). American Journal of Primatology 25:137–155. 10.1002/ajp.1350250302 31948180

[ajp22566-bib-0068] Schuett W , Dall SRX . 2009 Sex differences, social context and personality in zebra finches, *Taeniopygia guttata* . Animal Behaviour 77:1041–1050.

[ajp22566-bib-0069] Seyfarth RM , Cheney DL . 2012 The evolutionary origins of friendship. Annual Review of Psychology 63:153–177. 10.1146/annurev-psych-120710-100337 21740224

[ajp22566-bib-0070] Seyfarth RM , Silk JB , Cheney DL . 2012 Variation in personality and fitness in wild female baboons. Proceedings of the National Academy of Sciences 109:16980–16985. 10.1073/pnas.1210780109 PMC3479518 23027933

[ajp22566-bib-0071] Seyfarth RM , Silk JB , Cheney DL . 2014 Social bonds in female baboons: the interaction between personality, kinship and rank. Animal Behaviour 87:23–29.

[ajp22566-bib-0072] Sih A , Bell AM . 2008 Insights for behavioral ecology from behavioral syndromes. Advances in the Study of Behavior 38:227–281. 24991063 10.1016/S0065-3454(08)00005-3 PMC4075144

[ajp22566-bib-0073] Sih A , Bell AM , Johnson JC , Ziemba RE . 2004 Behavioral syndromes: an integrative overview. The Quarterly Review of Biology 79:241–277. 15529965 10.1086/422893

[ajp22566-bib-0074] Silk JB . 2007 Social components of fitness in primate groups. Science 317:1347–1351. 17823344 10.1126/science.1140734

[ajp22566-bib-0075] Smith BR , Blumstein DT . 2008 Fitness consequences of personality: a meta‐analysis. Behavioral Ecology 19:448–455.

[ajp22566-bib-0076] Smith BR , Blumstein DT . 2013 Animal personality and conservation biology: the importance of behavioral diversity In: Carere C, Maestripieri D, editors. Animal personalities: Behavior, physiology, and evolution. Chicago, IL: University of Chicago Press p 379–411.

[ajp22566-bib-0077] Stamps J , Groothuis TGG . 2010 The development of animal personality: relevance, concepts and perspectives. Biological Reviews 85:301–325. 19961473 10.1111/j.1469-185X.2009.00103.x

[ajp22566-bib-0078] Stevenson MF , Rylands AB . 1988 The marmosets, genus *Callithrix* In: Mittermeier RA, Rylands AB, Coimbra‐Filho AF, da Fonseca GAB, editors. Ecology and behavior of neotropical primates, Volume 2. Washington, D.C: World Wildlife Fund p 131–222.

[ajp22566-bib-0079] Stevenson MF , Poole TB . 1976 An ethogram of the common marmoset (*Callithrix jacchus jacchus*): General behavioural repertoire. Animal Behaviour 24:428–451. 820223 10.1016/s0003-3472(76)80053-x

[ajp22566-bib-0080] Stevenson‐Hinde J , Stillwell‐Barnes R , Zunz M . 1980a Subjective assessment of rhesus monkeys over four successive years. Primates 21:66–82.

[ajp22566-bib-0081] Stevenson‐Hinde J , Stillwell‐Barnes R , Zunz M . 1980b Individual differences in young rhesus monkeys: consistency and change. Primates 21:498–509.

[ajp22566-bib-0082] Stevenson‐Hinde J , Zunz M . 1978 Subjective assessment of individual rhesus monkeys. Primates 19:473–482.

[ajp22566-bib-0083] Suomi SJ . 1987 Genetic and maternal contributions to individual differences in rhesus monkey biobehavioral development In: Krasnegor NA, Bliss EM, Hofer MA, Smotherman WP, editors. Perinatal development: A psychobiological perspective. Orlando, FL: Academic Press p 397–419.

[ajp22566-bib-0084] Sussman AF , Ha JC , Bentson KL , Crockett CM . 2013 Temperament in rhesus, long‐tailed, and pigtailed macaques varies by species and sex. American Journal of Primatology 75:303–313. 23225368 10.1002/ajp.22104 PMC3581757

[ajp22566-bib-0085] Tabachnick BG , Fidell LS . 2007 Using multivariate statistics. 5th edition Boston: Pearson/Ally & Baconp 980.

[ajp22566-bib-0086] Uher J , Asendorpf JB , Call J . 2008 Personality in the behaviour of great apes: temporal stability, cross‐situational consistency and coherence in response. Animal Behaviour 75:99–112.

[ajp22566-bib-0087] Uher J , Addessi E , Visalberghi E . 2013a Contextualised behavioural measurements of personality differences obtained in behavioural tests and social observations in adult capuchin monkeys (*Cebus apella*). Journal of Research in Personality 47:427–444.

[ajp22566-bib-0088] Uher J , Werner CS , Gosselt K . 2013b From observations of individual behaviour to social representations of personality: developmental pathways, attribution biases, and limitations of questionnaire methods. Journal of Research in Personality 47:647–667.

[ajp22566-bib-0089] Uher J . 2008 Comparative personality research: methodological approaches. European Journal of Personality 22:427–455.

[ajp22566-bib-0090] Uher J . 2011a Individual behavioral phenotypes: an integrative meta‐theoretical framework. Why “behavioral syndromes” are not analogues of “personality”. Developmental Psychobiology 53:521–548. 21432848 10.1002/dev.20544

[ajp22566-bib-0091] Uher J . 2011b Personality in nonhuman primates: what can we learn from human personality psychology? In: Weiss A, King J, Murray L, editors. Personality and temperament in nonhuman primates. New York, NY: Springer p 41–76.

[ajp22566-bib-0092] Uher J , Asendorpf JB . 2008 Personality assessment in the great apes: comparing ecologically valid behavior measures, behavior ratings, and adjective ratings. Journal of Research in Personality 42:821–838.

[ajp22566-bib-0093] Voelkl B , Huber L . 2000 True imitation in marmosets. Animal Behaviour 60:195–202. 10973721 10.1006/anbe.2000.1457

[ajp22566-bib-0094] Webster MM , Ward AJW . 2011 Personality and social context. Biological Reviews 86:759–773. 21091603 10.1111/j.1469-185X.2010.00169.x

[ajp22566-bib-0095] Weinstein TAR , Capitanio JP , Gosling SD . 2008 Personality in animals In: John OP, Robins RW, Pervin LA, editors. Handbook of personality theory and research. New York: Guilford p 328–348.

[ajp22566-bib-0096] Weiss A , King JE , Perkins L . 2006 Personality and subjective well‐being in orangutans (*Pongo pygmaeus* and *Pongo abelii*). Journal of Personality and Social Psychology 90:501–511. 16594834 10.1037/0022-3514.90.3.501

[ajp22566-bib-0097] Wolf M , van Doorn GS , Leimar O , Weissing FJ . 2007 Life‐history trade‐offs favour the evolution of animal personalities. Nature 447:581–584. 17538618 10.1038/nature05835

[ajp22566-bib-0098] Zientek LR , Thompson B . 2007 Applying the bootstrap to the multivariate case: bootstrap component/factor analysis. Behavior Research Methods 39:318–325. 17695360 10.3758/bf03193163

