# Supplementary material for: Consistent inter‐individual differences in common marmosets (Callithrix jacchus) in Boldness‐Shyness, Stress‐Activity, and Exploration‐Avoidance
Source: Am J Primatol. 2016 Jun 10;78(9):961–73. doi: 10.1002/ajp.22566 (PMC4996331; doi:10.1002/ajp.22566)
Supplement: Supplementary file 1 — Supporting Information. [file AJP-78-961-s001.docx]

**Supplementary Electronic Material for:**

**Consistent inter-individual differences in common marmosets (*Callithrix jacchus*) in Boldness-Shyness, Stress-Activity and Exploration-Avoidance**

Šlipogor, V; Gunhold-de Oliveira, T; Tadić, Z; Massen, J.J.M & Bugnyar, T.

**INDEX**

Table S1. Details on the behavioral parameters..............................................................p2

Table S2. Temporal repeatability....................................................................................p3

Table S3. Contextual consistency....................................................................................p5

Table S4. PCA sample and bootstrap results...................................................................p6

Table S5. Best-fitting models (GLMMs)........................................................................p7

Table S6. Comparisons between tests and controls.........................................................p8

Disscussion - Controls.....................................................................................................p9

References.....................................................................................................................p11

**Table S1. Details on the behavioral parameters** coded: frequencies, durations and latencies.

| variable | description |
| --- | --- |
|  |  |
| enter latency | latency of subject to enter the experimental cage |
| body latency | latency of subject to be within one body length of the stimulus/object/food |
| touch latency | latency of subject to touch the stimulus/object/food |
| manipulation | subject actively manually or orally manipulates (i.e., touches, bites, licks or  scratches), smells and/or eats the stimulus/object/food; duration |
| stress behavior | subject scent marks inside of the cage, scratches itself, has pilo-erected fur [^*^, ^$^], defecates, urinates or manipulates the cage in a destructive manner; frequency |
| self-grooming | subject grooms itself; frequency |
| vigilance (mobbing/ aggression) calls | subject emits following calls: tsik, rapid tsik, tsik-egg, egg, cough, chatter, loud shrill [^#^,^+^]; frequency |
| contact calls | subject emits following calls: twitter, phee, see, trill [^#^]; frequency |
| food calls | subject emits following calls: chirp [^&^]; frequency |
| locomotion | subject walks, runs, climbs or jumps, with or without holding/manipulating/eating etc. stimulus/object/food (any movement in the experimental cage); duration |
| proximity | subject is in closest proximity to the experimental set-up (lower quarter of the experimental cage, i.e., virtual compartment diagonal to the subject's point of entrance); duration |
| distance | subject is furthest away from the experimental set-up (upper quarter of the experimental cage including the tunnel before the entrance door to the experimental cage, i.e., virtual compartment diagonal to the experimental set-up); duration |
| compartment alternations | number of times the subject changes virtual compartments; frequency |

^*^Barros et al. 2000, ^$^Stevenson & Poole 1976, ^#^Bezzera & Souto 2008, ^+^Epple 1968, ^&^Vitale et al. 2003

**Table S2.** **Temporal repeatability.** Summary of all behavioral variables and their temporal consistency as intra-class correlation (ICC 3,1) with 95% confidence intervals. The significantly repeatable behavioral variables are shown in bold.

| experiment | variable | ICC (3,1) | 95 % CI lower, upper | F, P-value |
| --- | --- | --- | --- | --- |
| GA^*^ | enter latency | 0.013 | -0.412, 0.433 | 1.027, 0.477 |
|  | body latency* | / | / | / |
|  | touch latency* | / | / | / |
|  | manipulation* | / | / | / |
|  | stress behavior | 0.264 | -0.179, 0.618 | 1.716, 0.118 |
|  | self-grooming | 0.110 | -0.328, 0.509 | 1.248, 0.312 |
|  | vigilance calls | -0.034 | -0.450, 0.394 | 0.934, 0.560 |
|  | **contact calls** | **0.466** | **0.053, 0.742** | **2.743, 0.014** |
|  | food calls | 0.327 | -0.111, 0.658 | 1.970, 0.069 |
|  | **locomotion** | **0.458** | **0.043, 0.738** | **2.688, 0.016** |
|  | proximity | -0.055 | -0.467, 0.377 | 0.896, 0.596 |
|  | distance | -0.247 | -0.606, 0.196 | 0.604, 0.866 |
|  | compartment alternations | 0.318 | -0.121, 0.653 | 1.933, 0.074 |
| tNF | enter latency | -0.030 | -0.447, 0.398 | 0.941, 0.553 |
|  | body latency | 0.011 | -0.413, 0.432 | 1.023, 0.480 |
|  | **touch latency** | **0.447** | **0.029, 0.731** | **2.614, 0.019** |
|  | **manipulation** | **0.472** | **0.061, 0.746** | **2.787, 0.013** |
|  | **stress behavior** | **0.542** | **0.154, 0.785** | **3.363, 0.005** |
|  | **self-grooming** | **0.369** | **-0.063, 0.685** | **2.171, 0.045** |
|  | **vigilance calls** | **0.506** | **0.106, 0.765** | **3.047, 0.008** |
|  | contact calls | 0.152 | -0.289, 0.540 | 1.358, 0.250 |
|  | food calls | 0.176 | -0.267, 0.557 | 1.427, 0.217 |
|  | **locomotion** | **0.447** | **0.030, 0.731** | **2.615, 0.019** |
|  | proximity | 0.194 | -0.250, 0.570 | 1.480, 0.194 |
|  | distance | -0.076 | -0.483, 0.358 | 0.859, 0.631 |
|  | **compartment alternations** | **0.593** | **0.227, 0.812** | **3.912, 0.002** |
| tNO | enter latency | 0.032 | -0.396, 0.448 | 1.065, 0.444 |
|  | body latency | 0.195 | -0.249, 0.570 | 1.483, 0.193 |
|  | touch latency | -0.107 | -0.507, 0.331 | 0.807, 0.682 |
|  | manipulation | -0.017 | -0.437, 0.409 | 0.967, 0.530 |
|  | stress behavior | 0.258 | -0.185, 0.613 | 1.694, 0.124 |
|  | **self-grooming** | **0.600** | **0.238, 0.816** | **4.000, 0.002** |
|  | vigilance calls | -0.015 | -0.435, 0.411 | 0.971, 0.526 |
|  | contact calls | 0.027 | -0.400, 0.445 | 1.056, 0.452 |
|  | food calls | -0.039 | -0.455, 0.390 | 0.924, 0.569 |
|  | **locomotion** | **0.438** | **0.018, 0.726** | **2.556, 0.021** |
|  | **proximity** | **0.786** | **0.545, 0.907** | **8.360, <0.001** |
|  | **distance** | **0.388** | **-0.042, 0.696** | **2.265, 0.037** |
|  | compartment alternations | 0.156 | -0.286, 0.543 | 1.368, 0.245 |
| tP | enter latency | 0.304 | -0.136, 0.644 | 1.875, 0.084 |
|  | **body latency** | **0.872** | **0.711, 0.946** | **14.614, <0.001** |
|  | touch latency | -0.038 | -0.454, 0.391 | 0.926, 0.567 |
|  | manipulation | -0.024 | -0.443, 0.402 | 0.952, 0.543 |
|  | stress behavior | -0.019 | -0.438, 0.407 | 0.962, 0.534 |
|  | self-grooming^#^ | / | / | / |
|  | **vigilance calls** | **0.691** | **0.380, 0.862** | **5.482, <0.001** |
|  | **contact calls** | **0.470** | **0.059, 0.745** | **2.775, 0.014** |
|  | food calls^#^ | / | / | / |
|  | locomotion | 0.254 | -0.189, 0.611 | 1.682, 0.127 |
|  | proximity | 0.309 | -0.131, 0.647 | 1.894, 0.081 |
|  | **distance** | **0.523** | **0.129, 0.774** | **3.193, 0.006** |
|  | **compartment alternations** | **0.722** | **0.431, 0.877** | **6.197, <0.001** |
| tFUR | enter latency | 0.282 | -0.160, 0.629 | 1.784, 0.102 |
|  | **body latency** | **0.780** | **0.533, 0.904** | **8.083, <0.001** |
|  | **touch latency** | **0.547** | **0.162, 0.788** | **3.416, 0.004** |
|  | **manipulation** | **0.558** | **0.177, 0.794** | **3.527, 0.003** |
|  | stress behavior | 0.264 | -0.179, 0.618 | 1.718, 0.117 |
|  | self-grooming^#^ | / | / | / |
|  | **vigilance calls** | **0.441** | **0.022, 0.728** | **2.576, 0.020** |
|  | contact calls | -0.059 | -0.470, 0.373 | 0.888, 0.603 |
|  | food calls | 0.227 | -0.216, 0.593 | 1.588, 0.154 |
|  | locomotion | 0.176 | -0.266, 0.557 | 1.427, 0.217 |
|  | **proximity** | **0.698** | **0.390, 0.865** | **5.618, <0.001** |
|  | **distance** | **0.767** | **0.510, 0.899** | **7.597, <0.001** |
|  | compartment alternations | 0.343 | -0.094, 0.668 | 2.042, 0.059 |

*Note that in the GA, body latency, touch latency and manipulation were not measured. In all other tests all variables were measured.

# Note that for these behavioral variables we were unable to compute the ICC values, due to too many zeros.

**Table S3. Contextual consistency** of the same behavioral variables across different tests. Significantly consistent variables are in bold.

| variable | experiments | cronbach's α | ICC | 95% CI lower, upper | F, P-value |
| --- | --- | --- | --- | --- | --- |
|  |  |  |  |  |  |
| stress behavior | tNF | n.a. | n.a. | n.a. | n.a. |
|  |  |  |  |  |  |
| **self-grooming** | tNF & tNO | 0.947 | 0.899 | 0.769, 0.958 | 18.882, <0.001 |
|  |  |  |  |  |  |
| manipulation | tNF & tFUR | -0.261 | -0.116 | -0.513, 0.323 | 0.793, 0.696 |
|  |  |  |  |  |  |
| contact calls | GA & tP | -0.223 | -0.100 | -0.502, 0.337 | 0.818, 0.671 |
|  |  |  |  |  |  |
| vigilance calls | tNF, tP & tFUR | 0.413 | 0.19 | -0.062, 0.491 | 1.703, 0.075 |
|  |  |  |  |  |  |
| body latency | tP & tFUR | 0.427 | 0.272 | -0.171, 0.623 | 1.746, 0.111 |
|  |  |  |  |  |  |
| touch latency | tFUR & tNF | 0.318 | 0.189 | -0.254, 0.566 | 1.466, 0.200 |
|  |  |  |  |  |  |
| **locomotion** | GA, tNF & tNO | 0.631 | 0.363 | 0.094, 0.634 | 2.712, 0.004 |
|  |  |  |  |  |  |
| **compartment alternations** | tNF & tP | 0.769 | 0.625 | 0.275, 0.829 | 4.338, <0.001 |
|  |  |  |  |  |  |
| **proximity** | tNO & tFUR | 0.655 | 0.487 | 0.080, 0.754 | 2.895, 0.011 |
|  |  |  |  |  |  |
| **distance** | tNO, tP & tFUR | 0.694 | 0.431 | 0.162, 0.683 | 3.268, <0.001 |

**Table S4. PCA sample and bootstrap results** across 1000 resamples. M(BR) - mean bootstrap results, SE – standard error. The results from the original PCA are italicized to distinguish the sample from the bootstrap results. In bold is the ratio of the mean bootstrap results and the standard error that is greater than or equal to 2.0 for each variable, salient to given components.

**Table S5. Best-fitting** **models (GLMMs)** performed on four factors obtained from the PCA analysis: a) Boldness-Shyness in Foraging, b) Boldness-Shyness in Predation, c) Stress-Activity, d) Exploration-Avoidance. Significant effects are indicated in bold.

| component | variable |  | F | (df1, df2) | β-coefficient | ± SE | P-value |
| --- | --- | --- | --- | --- | --- | --- | --- |
| a) Boldness-Shyness in Foraging | group (3) |  | 3.527 | (2, 11) |  |  | 0.066 |
|  |  | 1 |  |  | -1.961 | 2.118 |  |
|  |  | 2 |  |  | 5.054 | 2.471 |  |
|  | sex (male) |  | 1.353 | (1, 11) |  |  | 0.269 |
|  |  | female |  |  | -3.111 | 2.143 |  |
|  | age |  | 0.756 | (1, 11) |  |  | 0.403 |
|  | group*sex (3*male) |  | 0.338 | (2, 11) |  |  | 0.720 |
|  |  | 1*female |  |  | 1.140 | 1.394 |  |
|  |  | 1*male |  |  | 0 |  |  |
|  |  | 2*female |  |  | 0.255 | 1.173 |  |
|  |  | 2*male |  |  | 0 |  |  |
|  |  | 3*female |  |  | 0 |  |  |
|  | age*group (3) |  | 2.650 | (2, 11) |  |  | 0.115 |
|  |  | 1 |  |  | 0.074 | 0.324 |  |
|  |  | 2 |  |  | -0.512 | 0.263 |  |
|  | age*sex (male) |  | 1.997 | (1, 11) |  |  | 0.185 |
|  |  | female |  |  | 0.372 | 0.263 |  |
|  |  |  |  |  |  |  |  |
|  |  |  |  |  |  |  |  |
| b) Boldness-Shyness in Predation | age |  | 2.789 | (1, 13) |  |  | 0.119 |
|  | group*sex (3*male) |  | 2.192 | (5, 13) |  |  | 0.118 |
|  |  | 1*female |  |  | -3.121 | 1.877 |  |
|  |  | 1*male |  |  | -0.736 | 0.894 |  |
|  |  | 2*female |  |  | -5.446 | 2.155 |  |
|  |  | 2*male |  |  | 0.251 | 0.771 |  |
|  |  | 3*female |  |  | -3.006 | 1.811 |  |
|  | age*sex (male) |  | 4.119 | (1, 13) |  |  | 0.063 |
|  |  | female |  |  | 0.438 | 0.216 |  |
|  |  |  |  |  |  |  |  |
|  |  |  |  |  |  |  |  |
| c) Stress-Activity | group (3) |  | 3.399 | (2, 11) |  |  | 0.071 |
|  |  | 1 |  |  | -4.748 | 2.051 |  |
|  |  | 2 |  |  | -4.271 | 2.393 |  |
|  | sex (male) |  | 2.760 | (1, 11) |  |  | 0.125 |
|  |  | female |  |  | -3.444 | 2.075 |  |
|  | age |  | 3.785 | (1, 11) |  |  | 0.078 |
|  | group*sex (3*male) |  | 0.964 | (2, 11) |  |  | 0.412 |
|  |  | 1*female |  |  | -1.444 | 1.350 |  |
|  |  | 1*male |  |  | 0 |  |  |
|  |  | 2*female |  |  | 0.794 | 1.136 |  |
|  |  | 2*male |  |  | 0 |  |  |
|  |  | 3*female |  |  | 0 |  |  |
|  | age*group (3) |  | 2.976 | (2, 11) |  |  | 0.093 |
|  |  | 1 |  |  | 0.764 | 0.314 |  |
|  |  | 2 |  |  | 0.320 | 0.255 |  |
|  | age*sex (male) |  | 1.954 | (1, 11) |  |  | 0.190 |
|  |  | female |  |  | 0.357 | 0.255 |  |
|  |  |  |  |  |  |  |  |
|  |  |  |  |  |  |  |  |
| d) Exploration-Avoidance | **group (3)** |  | **26.544** | **(2, 15)** |  |  | **< 0.001** |
|  |  | 1 |  |  | -0.324 | 0.364 |  |
|  |  | 2 |  |  | -0.969 | 0.381 |  |
|  | **group*sex (3*male)** |  | **14.996** | **(3, 15)** |  |  | **< 0.001** |
|  |  | 1*female |  |  | 3.650 | 0.546 |  |
|  |  | 1*male |  |  | 0 |  |  |
|  |  | 2*female |  |  | 0.093 | 0.381 |  |
|  |  | 2*male |  |  | 0 |  |  |
|  |  | 3*female |  |  | -0.172 | 0.364 |  |
|  |  |  |  |  |  |  |  |

Reference groups are indicated in parenthesis.

**Table S6. Comparisons between tests and controls** of significantly repeatable variables. Variables that differ significantly between the test and control conditions are in bold.

| test & control | measure | z - score | P - value |
| --- | --- | --- | --- |
|  |  |  |  |
| tNF & cNF | **stress behavior** | **-2.075** | **0.038** |
|  | self-grooming | -0.966 | 0.334 |
|  | **manipulation** | **-2.242** | **0.025** |
|  | vigilance calls | -0.568 | 0.570 |
|  | **compartment alternations** | **-2.035** | **0.042** |
|  | touch latency | -1.008 | 0.313 |
|  | locomotion | -0.435 | 0.664 |
|  |  |  |  |
| tNO & cNO | self-grooming | -1.890 | 0.059 |
|  | **locomotion** | **-2.729** | **0.006** |
|  | proximity | -0.747 | 0.455 |
|  | distance | -0.052 | 0.958 |
|  |  |  |  |
| tP & cP | **body latency** | **-4.015** | **<0.001** |
|  | **vigilance calls** | **-4.015** | **<0.001** |
|  | **contact calls** | **-2.318** | **0.017** |
|  | **distance** | **-3.875** | **<0.001** |
|  | **compartment alternations** | **-3.702** | **<0.001** |
|  |  |  |  |
| tFUR & cFUR | **body latency** | **-3.621** | **<0.001** |
|  | **touch latency** | **-3.621** | **<0.001** |
|  | **manipulation** | **-3.920** | **<0.001** |
|  | **vigilance calls** | **-3.702** | **<0.001** |
|  | **proximity** | **-3.584** | **<0.001** |
|  | **distance** | **-3.25** | **<0.001** |

**Discussion - controls**

This study is one of the first studies on animal personality to use controls, which allow a careful interpretation of behavioral responses to novelty, predator and other contexts (as suggested by Carter, 2013). Like many other primates, common marmosets communicate with olfactory, visual and acoustic signals, but it seems that the latter, consisting of approximately 13 different calls [Bezerra & Souto, 2008], are the most important ones in arboreal habitats due to poor visibility [Altmann, 1967]. When marmoset groups detect a predator, they usually emit alarm calls, escape and hide, but sometimes they also approach and intensively mob it [Bezerra & Souto, 2008]. Mobbing is one tactic to reduce the chance of predation [Lorenz, 1931], and is often associated with loud calling. Mobbing alerts other unaware group members to the threat and ‘informs’ the predator it has lost its element of surprise. Many group-living species exhibit this behavior [e.g. Kobayashi, 1994; Owings & Coss, 1977], and it is thought to be advantageous in predator deterrence and risk assessment [cf. Carter et al., 2012; Dugatkin & Godin, 1992; FitzGibbon, 1994]. In this study, for instance, when subjects were exposed to a model of a predator in the test condition, they emitted significantly more vigilant and contact calls than when they were just exposed to leaves in the control condition. The time spent distant from the predator model was significantly longer in the test than in the control condition, as well as the latency to come within one body length to the predator model. This hints into the direction of risk-avoidance and shy behavior. Also, movement patterns scored as compartment alternations were significantly different between test and control conditions. In the foraging under risk tasks, subjects had to pass by a lychee fruit in order to obtain a food reward. Subjects needed significantly more time to do so and spent significantly less time manipulating the reward and being in proximity in the test than in the control condition. This again hints in the direction of shyness and risk-avoidance. This is in line with results from a study on arboreal ring-tailed lemurs where subjects reduced their food intake by not foraging on the ground when the predation risk was high [Sauther, 2002]. The number of vigilant calls that marmosets emitted was significantly higher in the test than in the control condition. This supports our prediction that a lychee fruit is a valid choice for a frightening stimulus, and in accordance with the aforementioned mobbing behavior of common marmosets. In the food test, subjects spent significantly more time manipulating the novel food than the familiar food, which contrasts with the findings of Vitale & Queyras [1997]. The fact that they did not find any differences between tests and controls may be due to the different measurement units used in their study (feeding and exploratory behavior sampling based on 10-second intervals, as opposed to total durations of behaviors). However, in a recent study by Carter et al. [2012], individual baboons (*Papio ursinus*) did not inspect the familiar food at all, but did inspect and manipulate the unfamiliar food. The frequency of stress behaviors as well as alternations between different compartments in the novel food condition were significantly different from the familiar (control) condition. This could be explained by an increase in excitement and interest in the novelty. Finally, in the novel object condition, even though the novel objects were very similar in size and shape to the familiar object, the duration of locomotion was significantly different between the test and the control. If the subjects were not excited and interested in novelty, similar movement and exploration patterns would be expected.

**REFERENCES**

Altmann SA. 1967. The structure of primate social communication. In: Altmann SA, editor. Social communication among primates. Chicago, IL: University of Chicago Press. p 325–362.

Bezerra BM, Souto A. 2008. Structure and usage of the vocal repertoire of *Callithrix jacchus*. International Journal of Primatology 29:671–701.

Carter A. 2013. On validity and controls in animal personality research: a comment on Galhardo et al. (2012). Biology Letters 9:20121080.

Carter AJ, Marshall HH, Heinsohn R, Cowlishaw G. 2012. How not to measure boldness: novel object and antipredator responses are not the same in wild baboons. Animal Behaviour 84:603–609.

Dugatkin L, Godin J. 1992. Prey approaching predators: a cost-benefit perspective. Annales Zoologici Fennici 29:233–252.

Epple G. 1968. Comparative studies on vocalization in marmoset monkeys (Hapalidae). Folia Primatologica 8:1–40.

FitzGibbon CD. 1994. The costs and benefits of predator inspection behaviour in Thomson’s gazelles. Behavioral Ecology and Sociobiology 34:139–148.

Kobayashi T. 1994. The biological function of snake mobbing by Siberian chipmunks: I. Does it function as a signal to other conspecifics? Journal of Ethology 12:89–95.

Lorenz K. 1931. Beiträge zur Ethologie sozialer Corviden. Journal für Ornithologie 79: 67–12.

Owings DH, Coss RG. 1977. Snake mobbing by California ground squirrels: adaptive variation and ontogeny. Behaviour 62:50–68.

Sauther ML. 2002. Group size effects on predation sensitive foraging in wild ring-tailed lemurs (*Lemur catta*). In Miller LE, editor. Eat or be eaten: predator sensitive foraging among primates. Cambridge, UK: Cambridge University Press. p 107–125.

Vitale A, Zanzoni M, Queyras A, Chiarotti F. 2003. Degree of social contact affects the emission of food calls in the common marmoset (*Callithrix jacchus*). American Journal of Primatology 59:21–28.

Vitale A, Queyras A. 1997. The response to novel foods in common marmoset (*Callithrix jacchus*): the effects of different social contexts. Ethology 103:395–403.
